# Recurrent Meningitis and Its Rare Association With Ligneous Conjunctivitis and Congenital Plasminogen Deficiency

**DOI:** 10.7759/cureus.44813

**Published:** 2023-09-07

**Authors:** Somayah A Alghubaishi, Muhammad Saeed, Fadi Abujamous, Hussam Osman, Badriah G Alasmari

**Affiliations:** 1 Pediatrics, Armed Forces Hospital - Southern Region, Khamis Mushait, SAU; 2 Pediatric Neurology, Armed Forces Hospital - Southern Region, Khamis Mushait, SAU; 3 Ophthalmology, Armed Forces Hospital - Southern Region, Khamis Mushait, SAU

**Keywords:** plasminogen, hypoplasminogenemia, congenital plasminogen deficiency, meningitis, conjunctivitis, plg gene, homozygous variant, ligneous conjunctivitis, pediatrics, genetics

## Abstract

This case report explores the rare association of recurrent meningitis, hydrocephalus, ligneous conjunctivitis, and congenital plasminogen deficiency in a term baby boy. Born at 39 weeks with a history of hydrocephalus, the neonate later developed ligneous conjunctivitis and a serious bout of meningitis. Genetic analyses confirmed a homozygous mutation in the *PLG *gene, indicative of congenital plasminogen deficiency. Despite intensive treatment, including a ventriculoperitoneal shunt for hydrocephalus and intravenous antibiotics for meningitis, the child succumbed to upper airway obstruction before reaching one year of age. This report underscores the medical complexity and severity of these interconnected conditions and advocates for further research to understand the interplay between them. Although this study is limited by its single-case nature and is not generalizable, it emphasizes the necessity for early recognition and a multidisciplinary treatment approach for better patient outcomes.

## Introduction

Hypoplasminogenemia (HPG), also referred to as congenital plasminogen deficiency (C-PLGD), is an uncommon multisystem disease distinguished by severe extracellular fibrinolysis. This process ultimately gives rise to the creation of solid pseudomembranes within the mucous membranes [1]. The prevalence of HPG is reported to be approximately 0.3-0.4% within the general population. In the European context, an estimated incidence of 1.6 cases per 106 inhabitants has been projected [2]. Drawing from the limited pool of epidemiological investigations conducted to date, the prevalence of HPG is approximated to be one case per 625,000 individuals [2].

Ligneous conjunctivitis is an uncommon eye condition that is characterized by chronic, recurrent conjunctivitis. This condition is often accompanied by the formation of thick nodular masses, pseudo-membrane, on the palpebral surfaces replacing the normal mucosa [3]. Ligneous conjunctivitis is inherited in an autosomal recessive pattern due to mutations in the *plasminogen* (*PLG*) gene or many other genes. It is associated with HPG, a type I PLG deficiency, as well [4].

Treating this disease is complex, and various therapeutic approaches have been utilized to address it [5]. In this report, we present a case of a term baby boy born at 39 weeks with a history of hydrocephalus to highlight the rare association between recurrent meningitis, hydrocephalus, ligneous conjunctivitis, and C-PLGD. Our aim is to shed light on the possible interplay between these conditions and emphasize the importance of recognizing this rare association. Furthermore, this report encourages further research into the complex relationships between these conditions.

## Case presentation

Background and ethical clearance

We present a case of a male neonate born at term, with a complex clinical course of recurrent meningitis, ligneous conjunctivitis, and congenital plasminogen deficiency. Ethical clearance for this case study was obtained from the family of the baby.

Family and antenatal history

The neonate was born at 39 weeks via cesarean section, necessitated by a previous cesarean section. APGAR scores were 8 and 9 at one and five minutes, respectively. The antenatal scan showed dilated lateral ventricles, a prominent third ventricle, and non-visualized septum lucidum, and the CT scan showed a dilated lateral ventricle with prominent lateral horn and absent septum pellucidum, suggestive of corpus callosum agenesis and supraoptic dysplasia (lobar holoprosencephaly). These antenatal findings were later confirmed by MRI and the case was reviewed by a senior neurosurgeon who suggested follow-up observation.

Physical examination and initial interventions

By the age of four months, the baby’s head circumference had increased from 34 cm at birth to 45 cm, necessitating admission to the neurosurgery department. At this time, a VP shunt was inserted, and at the same time, the baby developed ligneous conjunctivitis. Serum plasminogen was measured, and topical plasminogen medication along with fresh eye drops every four hours was administered. After three days of discharge, he developed meningitis presenting with fever, vomiting, and lethargy. A medical workup revealed the following: complete blood count: hemoglobin: 8 g/dL; platelet: 920/µL, C-reactive protein: 196.9 (units not specified, usually measured in mg/L). Cerebrospinal fluid analysis: white blood cell (WBC) count: 350/µL; red blood cells: 4/µL; polymorphs: 70% (percentage of total WBC count); lymphocytes: 30% (percentage of total WBC count); protein: 655 mg/dL; glucose: 1.2 mmol/L.

Culture showed *Staphylococcus epidermidis*. No significant findings were observed in an eye swab. The infection of the ventriculoperitoneal (VP) shunt was likely due to surgical contamination. The infection necessitated four weeks of intravenous antibiotics, which involved intravenous administration of vancomycin and ceftazidime, leading to full resolution of the meningitis. A change of shunt was performed, with culture results coming back to normal.

Ophthalmologic and cardiological findings

**Figure 1:**
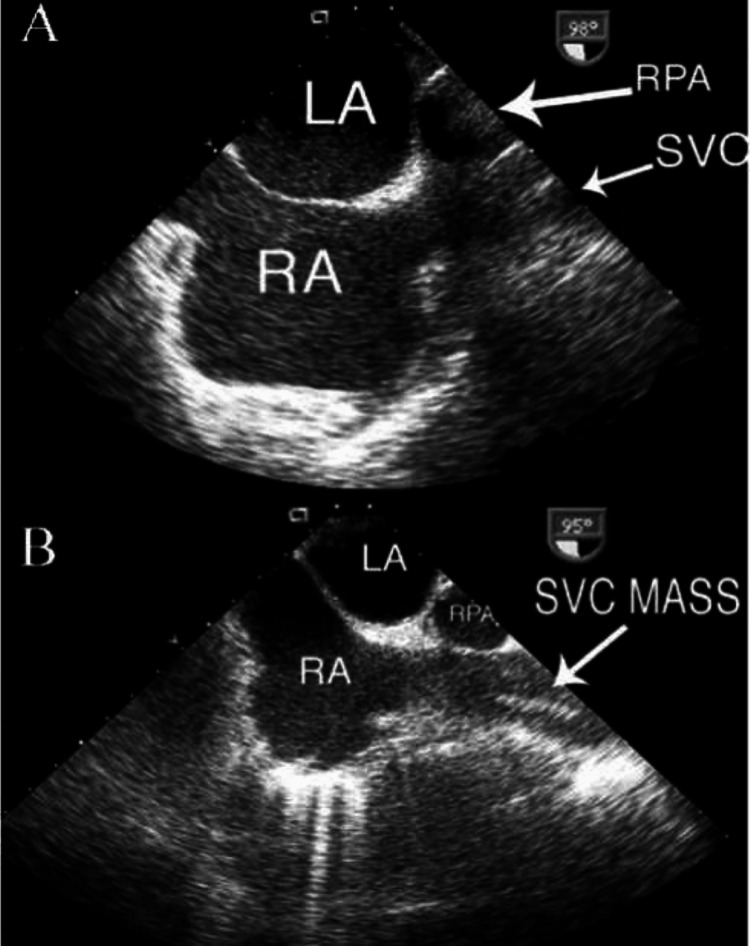
Echocardiography showed a large mass, most likely a thrombus, at the SVC-RA junction (arrow). SVC-RA: superior vena cava-right atrial

Although initially unremarkable, an ophthalmological examination in the fourth month revealed ligneous conjunctivitis characterized by hard, woody, and easily bleeding palpebral membranes. Echocardiography indicated a thrombus at the superior vena cava-right atrial junction, with extensions into various cardiac chambers, as shown in Figure 1.

Genetic and biochemical analyses

Whole exome sequencing (WES) confirmed homozygous variants in the *PLG *gene, indicative of autosomal recessive type 1 plasminogen deficiency. The test was conducted using Roche/KAPA sequence capture technology and sequenced on an Illumina system. Plasminogen levels were critically low at 15% (n = 75-150%).

Timeline and outcome

Despite intensive management, the patient tragically succumbed to upper airway obstruction before reaching one year of age. Radiological investigations and tandem mass spectrometry did not reveal additional anomalies. The significance of tandem mass spectrometry is that it rules out metabolic disorders, although in this case, it was not revealing. In summary, this case underscores the complexity and severity of C-PLGD and its rare but serious complications, including life-threatening thrombosis and recurrent meningitis.

## Discussion

Ligneous conjunctivitis is a rare form of chronic conjunctivitis that is more commonly observed in infants and young children than in older patients. Yellowish-white, fibrin-rich pseudomembranous lesions or red, hard masses that typically appear on the tarsal conjunctiva are characteristic of it. Most individuals with this condition tend to have a discharge of mucus from their affected eyes. This disease often leads to a common complication affecting the cornea, which may result in corneal scarring, vascularization, perforation, and reduced visual acuity [6]. Ligneous conjunctivitis typically affects both eyes and involves the palpebral conjunctiva [7].

Moreover, a case series sought to define the clinical and pathological context of a collection of ligneous conjunctivitis patients [8]. A total of 17 individuals were evaluated, most of whom were kids. The tarsal conjunctiva mostly presents with solid sessile or pedunculated membranous lesions. The larynx, voice cords, trachea, nose, vagina, cervix, and gingiva were also affected, presenting with identical and recurring lesions. Histopathologically, the lesions were subepithelial, eosinophilic, amorphous, and intermingled with acute and chronic inflammatory cells. The presence of amorphous material with immunoglobulins, albumin, and fibrin suggested leaks from blood vessels along with increased blood permeability. A few aberrant blood vessels with broad gaps between endothelial cells and thick multilaminar basement membranes were seen in the lesions when examined under the electron microscope. The illness might last anywhere from four months to 44 years. Six individuals experienced spontaneous remission following several recurrences [8].

During the ophthalmological examination at the age of four months, our patient presented with intriguing incidental findings. Bilateral tarsal lesions were observed, exhibiting a notable symmetry in appearance. A substantial and robust yellowish lesion, resembling a dense, hardened structure, firmly adhered to the tarsal conjunctiva. The diagnosis is based upon astute clinical examination and subsequent genetic tests, identifying a homozygous variant in the *PLG *gene, unveiling a rare and perplexing condition known as ligneous conjunctivitis. These woody, whitish-gray membranes, though visually unassuming, manifested an unusual tendency to bleed even with the gentlest of touches. A WES test was performed, which reported a homozygous variant c2095T>C p.(Cys699Arg) in the *PLG* gene. This variant causes autosomal recessive type 1 plasminogen deficiency. The plasminogen level was measured at 15%, which is well below the normal range of 75-150%.

Another previous case series was conducted by Klammt et al. (2011) to gain a better understanding of the genetic and clinical aspects related to severe HPG [9]. The study consisted of 23 cases and aimed to offer valuable insights into this condition. In 16 of 23 patients (about 70%), it was discovered that the *PLG* gene had homozygous or compound-heterozygous mutations, three of which were unique mutations that had never before been described (C166Y, Y264S, and IVS10-7T/G). It was also observed that the clinical symptoms of this group of 23 individuals showed greater rates of ligneous periodontitis, congenital hydrocephalus, and involvement of the female genital system when compared to the 79 previously described instances [9].

The relationship between HPG and ligneous conjunctivitis has been established due to decreased plasma plasminogen activity [6,7]. Wound healing involves inflammation, cell proliferation, migration, and matrix production. In ligneous conjunctivitis, the formation of granulation tissue in mucous membranes is impaired or arrested, resulting in fibrinogen being the main content of pseudomembranes. This material shows a significant deficiency in plasma plasminogen activity caused by recessive mutations in the *PLG *gene, which encodes plasminogen. Thus, pathologies found in ligneous conjunctivitis could be explained by the severe type 1 plasminogen deficiency [10].

Hydrocephalus is a serious medical condition that can be life-threatening. It is also commonly linked to ligneous conjunctivitis [11]. The cause of this condition is not yet fully understood. However, it is believed that the mechanism behind it may be similar to that of occlusive hydrocephalus. Fibrin deposition in the cerebral ventricular system damages the circulation of cerebrospinal fluid in the aqueduct, leading to fluid retention [12].

Comorbid ligneous conjunctivitis and congenital occlusive hydrocephalus have been reported in children [10-13]. Our case report highlights the association between congenital hydrocephalus and ligneous conjunctivitis. This connection has been previously reported in cases where hydrocephalus was treated with a cerebral shunt in infancy.

Currently, there is no widely accepted treatment protocol for ligneous conjunctivitis. However, topical plasminogen or heparin eye drops, topical or systemic fresh frozen plasma, and surgical excision of ligneous pseudomembranes were previously reported to be used as individual treatment modalities for ligneous conjunctivitis, most often with minor or sporadic success [14].

In our case, as part of the daily treatment plan, the patient received fresh frozen plasma to restore plasma plasminogen level, topical steroids to reduce inflammation, heparin eye drops to prevent blood clots, and artificial tear drops to alleviate dryness. Despite the severity of the condition, no systemic therapy was deemed necessary. Regarding the management of hydrocephalus, VP shunts have been a critical and commonly used treatment approach. It involves the insertion of a catheter that runs from the ventricle of the brain to the peritoneal cavity in the abdomen. VP shunts play a pivotal role in diverting excess cerebrospinal fluid from the brain’s ventricles to the peritoneal cavity, effectively alleviating intracranial pressure and mitigating associated symptoms. However, one of the challenges associated with VP shunts is the occurrence of frequent occlusions, necessitating shunt repairs [15].

In our patient, hydrocephalus was suspected prenatally and confirmed postnatally through imaging, with subsequent identification of PLG deficiency following the development of ligneous conjunctivitis. The VP shunt experienced two occlusions, leading to recurrent positive culture meningitis. Notably, even in individuals with extremely low plasminogen levels, the risk of venous thrombosis does not appear to be elevated in both homozygous and heterozygous cases. It is theorized that while the typical intravascular plasmin-based pathway of fibrinolysis is interrupted, compensation occurs through an enhanced fibrin/fibrinogen clearance mechanism involving alternative serine proteases or monocytoid cells [16].

This case report makes a substantial scientific contribution by elucidating the concurrent occurrence of recurrent meningitis, ligneous conjunctivitis, hydrocephalus, and C-PLGD within a single patient. It spotlights the critical importance of early recognition of this rare association, potentially enabling timely interventions and improved clinical outcomes. Moreover, it supports the use of a multidisciplinary approach to manage patients and tackle the complex challenges presented by these conditions when they occur together. Furthermore, this report is a driving force for additional scientific research, providing the possibility for improved therapeutic methods and preventative strategies.

Limitations

This is a single case report, and thus the findings are not generalizable. Potential biases include observational data and limited genetic testing of family members for the identified *PLG *variant.

## Conclusions

Ligneous conjunctivitis typically presents as a clinical symptom of C-PLGD. This case report underscores its significance in the context of congenital occlusive hydrocephaly, particularly in neonates with ligneous conjunctivitis or a family history of this condition. It also highlights the importance of considering *PLG *deficiency in occlusive hydrocephaly cases and emphasizes the importance of comprehensive assessments and genetic evaluations in neonates and individuals with relevant clinical presentations or familial predispositions. This broader clinical perspective emphasizes the need for early diagnosis and tailored management strategies.
